# The Association Between Changes in Insulin Sensitivity and Consumption of Tobacco and Alcohol in Young Adults: Ordinal Logistic Regression Approach

**DOI:** 10.7759/cureus.942

**Published:** 2016-12-24

**Authors:** Dragan Skropanic, Gudeta Fufaa, Bin Cai

**Affiliations:** 1 Mathematics and Statistics Department, Western Wyoming Community College; 2 School of Health Sciences, Walden University

**Keywords:** diabetes, insulin resistance, insulin sensitivity, ordinal logistic regression, quicki, young adults

## Abstract

**Context:**

Reduced insulin sensitivity is one of the traditional risk factors for chronic diseases such as type 2 diabetes. Reduced insulin sensitivity leads to insulin resistance, which in turn can lead to the development of type 2 diabetes. Few studies have examined factors such as blood pressure, tobacco and alcohol consumption that influence changes in insulin sensitivity over time especially among young adults.

**Purpose:**

To examine temporal changes in insulin sensitivity in young adults (18-30 years of age at baseline) over a period of 20 years by taking into account the effects of tobacco and alcohol consumptions at baseline. In other words, the purpose of the present study is to examine if baseline tobacco and alcohol consumptions can be used in predicting lowered insulin sensitivity.

**Method:**

This is a retrospective study using data collected by the Coronary Artery Risk Development in Young Adults (CARDIA) study from the National Heart, Lung, and Blood Institute. Participants were enrolled into the study in 1985 (baseline) and followed up to 2005. Insulin sensitivity, measured by the quantitative insulin sensitivity check index (QUICKI), was recorded at baseline and 20 years later, in 2005. The number of participants included in the study was 3,547. The original study included a total of 5,112 participants at baseline. Of these, 54.48% were female, and 45.52% were male; 45.31% were 18 to 24 years of age, and 54.69% were 25 to 30 years of age. Ordinal logistic regression was used to assess changes in insulin sensitivity.

Changes in insulin sensitivity from baseline were calculated and grouped into three categories (more than 15%, more than 8.5% to at most 15%, and at most 8.5%), which provided the basis for employing ordinal logistic regression to assess changes in insulin sensitivity. The effects of alcohol and smoking consumption at baseline on the change in insulin sensitivity were accounted for by including these variables in the model.

**Results:**

Daily alcohol consumption (ml/day) at baseline was not associated with changes in insulin sensitivity (OR = 0.998, 95% CI 0.995-1.001), while the number of cigarettes consumed per day at baseline was statistically significantly associated with changes in insulin sensitivity (OR = 1.016, 95% CI 1.007-1.025). Covariates such as age (OR = 1.05, 95% CI 1.031-1.071), mean arterial blood pressure (OR = 0.986, 95% CI 0.977-0.994), body-mass index (OR = 0.951, 95% CI 0.936-0.965), race (OR = 0.840, 95% CI 0.735-0.960), and sex (OR = 0.561, 95% CI 0.483-0.652) were significantly associated with changes in insulin sensitivity.

**Conclusion:**

After adjusting for relevant covariates, the daily tobacco consumption at baseline was independently associated with changes in insulin sensitivity. But we were not able to replicate the association between daily alcohol consumption at baseline and changes in insulin resistance reported by other studies. Further studies in different populations and settings are warranted to examine the association between alcohol consumption and changes in insulin resistance.

## Introduction

Insulin sensitivity is a measure of how fast a body can process glucose whereas insulin resistance is a condition in which a body is unable to efficiently process glucose. Lowered insulin sensitivity may lead to insulin resistance, which in turn leads to elevated levels of serum glucose and eventually to type 2 diabetes [1]. The most important risk factors for insulin resistance are overweight and obesity, genetics (family history), older age, and elevated blood pressure [2]. Studies that investigated the progression from lowered insulin sensitivity to insulin resistance and type 2 diabetes using longitudinal data are limited [1-2].

In this study, percent change of insulin sensitivity and its association with baseline tobacco and alcohol consumptions is investigated after having accounted for relevant co-variates. The association between insulin sensitivity and tobacco consumption has been studied in recent years, and some epidemiological characteristics were obtained although the physiological properties of this association have not been fully explained or understood [1, 3]. A study completed by Persson and colleagues in Sweden that involved men aged 35-56 provided evidence that heavy smoking was associated with a higher incidence of insulin resistance (OR = 2.7, 95% CI 1.3-5.5) [4]. Some of the other studies focused on type 2 diabetes and its dependence on tobacco consumption. A meta-analysis of 25 prospective studies showed that high cigarettes consumption (defined as 20 cigarettes or more per day) placed the participants at a much higher risk for type 2 diabetes than those with lower cigarette consumption per day (combined RR = 1.61, 95% CI 1.43-1.80) [5]. Another study by Zhang and colleagues showed that active smokers were at an increased risk for type 2 diabetes (RR = 1.39, 95% CI 1.17-1.64) [6]. The association can be explained by the fact that the tobacco toxicity in the blood stream may interfere with the beta-cells’ normal functioning, which in turn can trigger disruptions in normal functioning of the glucose transporters at the cellular level [5-7].

The nature of the association between insulin resistance and alcohol consumption is quite different. This has been explained by the role of alcohol in lowering the high density lipoprotein (HDL) cholesterol levels and reducing the inflammation at the cellular level [8-9]. A large prospective study involving 38,031 men indicated that moderate alcohol consumption (7.5 g per day) may lower the risk of type 2 diabetes over four years by as much as 22% (HR = 0.78, 95% CI 0.60-1.00) [9]. Those who consumed 7.5 g to 15 g of alcohol per day also exhibited a lowered risk (HR = 0.89, 95% CI 0.83-0.96), while those who consumed more than 15 g of alcohol per day exhibited neither lower nor higher risk (HR = 0.99, 95% CI 0.95-1.02) [9]. In yet another study, it was shown that moderate alcohol consumption may reduce the risk of type 2 diabetes in men by as much as 52% (HR = 0.48, 95% CI 0.28-0.77) and in women by as much as 19% (HR = 0.81, 95% CI 0.33-1.96) [10].

The present study used the data collected in the Coronary Artery Risk Development in (Young) Adults (CARDIA). CARDIA was initiated in 1984 by recruiting Black and White young adults aged 18-30 at four different field centers: Birmingham, Alabama; Chicago, Illinois; Minneapolis, Minnesota; and Auckland, California [11]. The original purpose of the study, which is still ongoing, was to monitor changes in cardiovascular health indicators over a long period of time. The secondary purpose of the study was to follow the participants’ insulin health as well as to identify possible occurrence of prediabetes and diabetes [11-12]. The baseline characteristics were collected in 1985 (year 1). The insulin level was recorded in year 1 (1985) and year 20 (2005). The quality of the data was assured by the standard steps and procedures that the principal investigators followed in recruitment of the participants, securing confidentiality of the data, and proper management and storage of the data [11-12].

For this study, insulin sensitivity is measured by the quantitative insulin sensitivity check index (QUICKI), which has been validated as a reliable measure of insulin sensitivity [13]. In particular, using the CARDIA data set, QUICKI is calculated as:

$QUICKI=\frac{1}{ln(IG)}=\frac{1}{lnI+lnG}$

where I is the insulin level (measured in μmol/ml) and G is the glucose level (measured in mg/dl). QUICKI’s sensitivity and specificity have been estimated at 80% and 60%, respectively [13]. One can show that QUICKI is a decreasing function of insulin and glucose, meaning that as insulin and glucose increase, the QUICKI decreases [1]. The percent change in insulin sensitivity depends on the QUICKI readings in 1985 and 2005, and is defined as follows [1]:

$\frac{QUICKI(1985)-QUICKI(2005)}{QUICKI(1985)}100$

The traditional cut-off points for QUICKI are as follows: QUICKI of 0.310 or less is an indication of the presence of insulin intolerance and/or type 2 diabetes; QUICKI of 0.366 or more, on the other hand, may be an indication of absence of insulin intolerance and/or type 2 diabetes [1, 14]. Levels between 0.310 and 0.357 may indicate the presence of insulin intolerance and prediabetes, while values between 0.357 and 0.366 may be an indication of absence of insulin intolerance [12].

## Materials and methods

We sought to assess the changes in insulin sensitivity in a longitudinal setting. Ordinal logistic regression models were used wherein the percent change in insulin sensitivity was used as the main outcome variable. Insulin sensitivity was measured by the QUICKI. The study utilized the data collected by the CARDIA, and is based on year 1 (1985) and year 20 (2005) data. Main explanatory variables were alcohol and tobacco consumptions. Corresponding odds ratios and the associated confidence intervals were reported for these two main explanatory variables as well as for other variables used in the regression model, such as creatinine level, age, mean arterial blood pressure, body-mass index (BMI), serum cholesterol, race, and sex (gender).

The percent change in insulin sensitivity depends on the QUICKI readings in 1985 and 2005 and is defined as follows:

$\frac{QUICKI(1985)-QUICKI(2005)}{QUICKI(1985)}100$

To categorize the percent change in insulin sensitivity, the cut-off points of 8.5% and 15% were used. The first cut-off point of 8.5% was discovered to be a significant mean difference, while the second cut-off point is calculated as:

$\frac{0.357-0.310}{0.310}100$

These cut-off points will provide the necessary categories in the dependent (outcome) variable. In particular, the first category consists of percent change values of less than 8.5%, the second category of percent change values of 8.5% to at most 15%, and the third category of percent change values of more than 15%.

STATA 13.0 (College Station, TX) software was used to code the variables, calculate QUICKI, obtain the descriptive statistics, and the results and diagnostics for the ordinal logistic regression models. Ordinal logistic regression model was used to evaluate the impact of independent variables on the ordinal categories of percent change in insulin sensitivity. Brand test was used to evaluate the parallel regression assumption. Additional tests, such as paired t-test for mean difference, one-way analysis of variance, and chi-square tests were also performed by STATA 13.0.

## Results

Initially, 5,112 participants were recruited into the study. Of these, 2,475 (48.42%) were White and 2,637 (51.58%) were African American. Moreover, 2,785 (54.48%) were female and 2,327 (45.52%) were male. The participants’ average age at baseline was 24.8 years with the standard deviation of 3.6 years. The average BMI was 24.4 kg/m^2^ with the standard deviation of 4.81 kg/m^2^. Basic descriptive statistics for continuous demographic and biometric variables at baseline are provided in Table 1.

**Table 1:** Descriptive Statistics for Continuous Variables: Demographic and Biometric Characteristics at Baseline SD = standard deviation, Q1 = 25th percentile, Q3 = 75th percentile, IQR = inter-quartile range (Q3-Q1), MAP = mean arterial blood pressure, MIN = smallest observation, MAX = largest observation

Variable (Baseline)	Number of Valid Observations	Mean	*SD*	Median	Q1	Q3	IQR	MIN	MAX
Age (years)	5,112	24.76	3.63	25.00	22.00	28.00	6.00	18.00	30.00
Weight (kg)	5,096	70.95	15.52	68.67	59.42	79.51	20.09	43.00	127.00
Height (cm)	5,099	170.35	9.27	170.00	163.00	177.50	14.50	149.50	193.00
BMI (kg/m^2^)	5,095	24.44	4.81	23.44	21.15	26.42	5.27	17.03	45.85
Alcohol consumption (ml/day)	5,091	12.08	21.95	4.77	0.00	14.70	14.70	0.00	335.02
Creatinine (mg/dl)	5,047	1.04	0.35	1.00	0.90	1.20	0.30	0.50	15.40
Glucose (mg/dl)	5,031	82.61	16.31	81.00	77.00	87.00	10.00	37.00	457.00
Insulin (μu/ml)	5,042	10.86	7.98	8.75	6.10	13.00	6.90	2.10	96.90
Tobacco consumption (number of cigarettes smoked per day)	5,112	4.00	7.94	0.00	0.00	5.00	5.00	0.00	75.00
Systolic blood pressure (mmHg)	5,112	110.42	10.95	110.00	103.00	118.00	15.00	75.00	167.00
Diastolic blood pressure (mmHg)	5,111	68.59	9.62	68.00	62.00	75.00	13.00	0.00	113.00
MAP	5,111	82.54	9.02	82.00	76.67	88.33	11.66	30.33	125.67
Cholesterol (mg/dl)	5,063	176.76	33.47	174.00	153.00	197.00	44.00	72.00	342.00

When it comes to alcohol consumption, the average was 12.08 ml/day while the standard deviation was 21.95 ml/day. The average tobacco consumption was four cigarettes/day with the standard deviation of 7.94 cigarettes/day. Stratified statistics for daily tobacco and alcohol consumptions are given in Tables 2-3 below.

**Table 2:** Descriptive Statistics for Daily Tobacco Consumption (cigarettes/day) According to Rank of Change in Insulin Sensitivity The non-zero tobacco consumption suggests that at least on some occasions all of the subjects included in the study did consume some tobacco.

Rank for Percent Change in Insulin Sensitivity	Number of Observations	Minimum	Maximum	Mean	Std Dev
0	858	0	71	3.292	7.562
1	859	0	52	2.725	6.804
2	859	0	60	3.608	7.780
3	858	0	60	4.623	8.788

**Table 3:** Descriptive Statistics for Daily Alcohol Consumption (ml/day) According to Rank of Change in Insulin Sensitivity The non-zero alcohol consumption suggests that at least on some occasions all of the subjects included in the study did consume some alcohol.

Rank for Percent Change in Insulin Sensitivity	Number of Observations	Minimum	Maximum	Mean	Std Dev
0	858	72	330	178.962	33.604
1	859	86	332	177.681	33.183
2	859	78	342	177.040	33.698
3	858	93	303	176.096	33.053

Insulin sensitivity levels from 1985 and 2005 were also compared. The results are summarized in Table 4 and Table 5 below.

**Table 4:** Descriptive Statistics for Insulin Sensitivity

Variable	Number of Valid Observations	Mean	*SD*	Median	Q1	Q3	IQR	MIN	MAX
Insulin sensitivity (QUICKI) in 1985	5,008	0.153	0.014	0.15	0.14	0.16	0.02	0.10	0.20
Insulin sensitivity (QUICKI) in 2005	3,449	0.140	0.012	0.14	0.13	0.15	0.02	0.10	0.21
Percent change in insulin sensitivity	3,434	8.27	9.30	8.79	2.80	14.33	11.53	-36.24	37.14

**Table 5:** Insulin Sensitivity from 1985 to 2005 The mean difference in insulin sensitivity was statistically significant (paired t = 52.822, P < 0.001).

Variable	Number of Observations	Mean	Std Dev	95% Confidence Interval	
Insulin sensitivity in 1985	3,434	0.153	0.014	0.153	0.154
Insulin sensitivity in 2005	3,434	0.140	0.012	0.139	0.140
Difference*	3,434	0.013	0.015	0.013	0.014

The average cholesterol level was 176.76 mg/dl with the standard deviation of 33.47 mg/dl. The average QUICKI at baseline was 0.153 with the standard deviation of 0.014; the average QUICKI in 2005 was 0.140 with the standard deviation of 0.012. These results were also reported in the previous study [1].

The results of our analyses indicate that alcohol consumption was not a statistically significant predictor (OR = 0.998, 95% CI 0.995-1.001, p = 0.198). Despite this result, alcohol consumption should be monitored as it may affect overall long-term health [10]. Moreover, tobacco consumption was statistically significant (OR = 1.016, 95% CI 1.008-1.025, p < 0.001).

Further results of our investigation revealed that mean arterial blood pressure (OR = 1.022, 95% CI 1.012-1.033, p < 0.001), BMI (OR = 1.063, 95% CI 1.041-1.086, p < 0.001), race (OR = 1.283, 95% CI 1.082-1.521, p < 0.01), and sex (OR = 1.792, 95% CI 1.470-2.184, p < 0.001) are significant predictors of the risk of insulin resistance.

Collinearity diagnostics indicate that there were no issues as the maximum variance inflation factor was 1.30 and the minimum variance inflation factor was 1.05.

The one-way analysis of variance results indicated that the age was significantly different among the three categories of the percent change in insulin sensitivity (F = 7.74, p < 0.001). Similarly, mean arterial blood pressure was another significant factor (F = 6.40, p = 0.002). Furthermore, BMI showed significant difference among the three categories (F = 22.75, p < 0.001). Similarly, tobacco consumption (the number of cigarettes per day) was significantly different among the three categories of the percent change in insulin sensitivity (F = 11.24, p < 0.001).

The results of the full ordinal logistic regression model are summarized in Table 6.

**Table 6:** Results from the Full Regression Model (based on n = 3,394) Tobacco = daily tobacco consumption, MAP = mean arterial blood pressure, Alcohol = daily alcohol consumption, BMI = body-mass index

Baseline Variable	Odds Ratio	p value	95% Confidence Interval	
Creatinine	0.958	0.772	0.757	1.212
Tobacco	1.016	<0.001	1.008	1.025
Age	1.051	<0.001	1.032	1.071
MAP	0.986	0.001	0.979	0.994
Alcohol	0.998	0.198	0.995	1.001
BMI	0.951	<0.001	0.936	0.965
Cholesterol	0.999	0.418	0.997	1.001
Race	0.840	0.011	0.735	0.960
Sex	0.561	<0.001	0.483	0.652

The results in Table 6 were based on the 3,394 valid cases, meaning that any subjects with missing values were not included in the model. The results of the Brant test indicated that the model satisfied the parallel regression assumption (overall χ2 = 17.65, p = 0.039, df = 9). Removal of statistically insignificant variables in the reduced regression model did not yield significantly different odds ratios for the statistically significant variables.

## Discussion

This study shows that number of cigarettes consumed per day increased the risk of lowered insulin sensitivity by 1.6% (OR = 1.016, 95% CI 1.008-1.025, p < 0.001). In other words, one cigarette increase per day would increase this risk by 1.6% on average. This finding corroborates current literature and the previous study in which the binary logistic regression model was used to obtain the odds ratios [1]. However, the researchers did not find a statistically significant association between alcohol consumption and change in insulin sensitivity (OR = 0.998, 95% CI 0.995-1.001, p = 0.198), which is different from the report in the current literature. Alcohol consumption has been reported to reduce the occurrence of lowered insulin sensitivity, although heavy alcohol consumption presents neither a benefit nor a threat to lowered insulin sensitivity [1, 9].

In the present study, baseline cholesterol level turned out to be a non-significant factor (OR = 0.999, 95% CI 0.997-1.001, p = 0.418), which does not corroborate the findings in the current literature. This may be due to the fact that baseline cholesterol readings were taken when the study participants were young adults. It may also suggest temporal changes in serum cholesterol may be a more meaningful indicator. Baseline serum creatinine was not a significant factor (OR = 0.958, 95% CI 0.757-1.212, p = 0.722) either, which might be due to the same reason as baseline cholesterol level. Studies exploring the association between serum creatinine and lowered insulin sensitivity and type 2 diabetes are rare. Only one study provided the results that lowered creatinine levels are associated with the risk of type 2 diabetes (OR = 1.69, 95% CI 1.32-2.15) [15].

Other results of this study may be of interest for current and future investigations. In particular, the investigators discovered that sex is a statistically significant predictor (OR = 0.561, 95% CI 0.483-0.652, p < 0.001), meaning that men were at a higher risk than women to exhibit a lowered insulin sensitivity. Other statistically significant predictors were mean arterial blood pressure (OR = 0.986, 95% CI 0.979-0.994, p = 0.001), BMI (OR = 0.951, 95% CI 0.936-0.965, p < 0.001), and race (OR = 0.840, 95% CI 0.735-0.960, p = 0.011). Results for race indicated that Whites, when compared to Blacks, were at a reduced risk of developing lowered insulin sensitivity. This may have serious implications for present and future public health intervention programs and plans.

There are several limitations that need to be mentioned. First, the study’s participants come only from four urban areas of the United States: Birmingham, Alabama; Chicago, Illinois; Minneapolis, Minnesota; and Oakland, California. Despite the fact that geographical variability is represented, rural populations were not represented. Second, only two racial groups, African Americans and Whites, were included in the study. Furthermore, data for many participants were missing due to the loss of follow-ups, which may have affected the results. Finally, the data for the independent variables come from the baseline only without considering the potential changes over the 20-year period.

The strengths of the study include a large sample size, longitudinal nature of the data, geographic diversity of the participants, as well as the measurement of the bio-indicators over a long period of time with five-year visit intervals.

## Conclusions

The results of the ordinal logistic regression showed that daily tobacco consumption is a significant predictor of changes in insulin regression, while daily alcohol consumption is not. In particular, the risk of lowered insulin sensitivity was increased by 1.6% (OR = 1.016, 95% CI 1.008-1.025) with every additional cigarette smoke per day. On the other hand, alcohol consumption was not a significant predictor of change in insulin resistance (OR = 0.998, 95% CI 0.995-1.001). Nonetheless, alcohol consumption should continue to be monitored as it has been linked to many other health conditions and illnesses.

Being able to predict future reduced insulin sensitivity using baseline data is challenging. Our study results showed that smoking, as well as race, sex, BMI, and arterial blood pressure can be used in predicting insulin resistance. Therefore, the implications for health care and public health are significant. Intervention programs can be designed to modify life style habits in order to reduce the risk for development of insulin resistance, and eventually, type 2 diabetes.

In future studies one may consider the effect of overall change in the independent variables (i.e. time-dependent modeling such as in survival analysis) over the designated time to better describe the association.
